# Analysis of EGFR Mutation Status in Algerian Patients with Non-Small Cell Lung Cancer

**DOI:** 10.31557/APJCP.2021.22.4.1063

**Published:** 2021-04

**Authors:** Mohamed Lahmadi, Leila Beddar, Amira Lyna Rouibah, Ali Boumegoura, Houda Boufendi, Asma Temim, Anissa Nini, Feriel Sellam, Dalila Satta

**Affiliations:** 1 *Department of Animal Biology, University of Mentouri Brothers Constantine 1, Constantine, Algeria. *; 2 *Biotechnology Research Center (CRBt), Constantine, Algeria. *; 3 *Department of Anatomical Pathology, Ben-Badis Hospital, Faculty of Medicine University of Constantine 3, Constantine, Algeria. *

**Keywords:** Non-small cell lung cancer, Mutation, EGFR gene, Algeria

## Abstract

**Background and objective:**

Epidermal growth factor receptor (EGFR) mutation status is used as a predictive biomarker for the tyrosine kinase inhibitors therapy in non-small cell lung cancer (NSCLC). The incidence of EGFR mutations appears to vary according to ethnic and geographical backgrounds. This retrospective study aimed to investigate the EGFR mutation status in Algerian NSCLC patients and its association with clinicopathological features.

**Methods:**

We examined the presence of EGFR mutations (Exons 19-21) in 58 unselected NSCLC samples using PCR followed by direct sequencing.

**Results:**

The present study included 53 (91.4%) men and 5 (8.6%) women, with a median age of 59 (ranging from 44 to 94 years old). EGFR mutations were detected in 23 patients, with an overall rate of 39.6%. There were 21 (91.3%) cases with the exon-21 L585R single mutation and two (8.7%) with dual mutations of exon-19 deletions and L585R. EGFR mutations were more frequently found in patients with confirmed adenocarcinoma (14/27, 51.8%) than in non-adenomatous NCSCL subtypes (3/14, 21.4%; p=0.03). Furthermore, early stages of the disease were significantly associated with a higher rate of EGFR mutations (14/27, 51.8%) compared with those at advanced stage (5/21, 23.8%; p=0.02). There were no significant differences in EGFR mutation frequency by age, gender, or smoking status.

**Conclusion:**

We found that Algerian NSCLC patients exhibited a high rate of EGFR mutations, which was quite similar to that in Asians population rather than Caucasian patients. Thus, TKI-based treatments may be more beneficial for Algerian patients with NSCLC. Further studies using a large number of patients are required to confirm our preliminary findings.

## Introduction

Lung cancer remains the leading cause of cancer-related mortality worldwide. According to the latest global cancer statistics, it is responsible for an estimated 1.7 million deaths (18.4% of the total cases) in 2018, and its 5-year survival rate is still at a low level (Bray et al., 2018). Lung cancer is the most commonly diagnosed cancer among males in the Northwest African or Maghreb region, including Algeria (nearly 13.6% of the total cases), Morocco (22.9%), Tunisia (20.2%), and Libya (18.9%). In Algeria, lung cancer is the leading cause of cancer-related death in men, and ranked second in both sexes after breast cancer, with a global age-standardized mortality rate of 10 per 100.000 (https://gco.iarc.fr/today/).

Non-small cell lung cancer (NSCLC) accounts for approximately 85% of all lung cancer cases, which is further classified as adenocarcinoma, squamous cell carcinoma, large cell carcinoma, and other rare subtypes (Travis et al., 2015). The discovery of several targetable genetic alterations in NSCLC and subsequent development of targeted therapies for these patients’ subsets have revolutionized the diagnosis and treatment of advanced NSCLCs. The epidermal growth factor receptor (EGFR) and anaplastic lymphoma kinase (ALK) are the most clinically relevant oncogenic drivers in NSCLC (Gaughan and Cost, 2011; Levy et al., 2012). EGFR mutations have been proved to be relevant to NSCLC adenocarcinomas as a predictive biomarker for the tyrosine kinase inhibitors (TKIs) therapy in EGFR-mutant patients. The activating EGFR mutations involve four exons (18–21) within the tyrosine kinase domain, including the largest portion in-frame deletions of exon 19 (Ex19del) and L858R missense point mutation in exon 21 (Lynch et al., 2004; Pao et al., 2004; Murray et al., 2008). However, tumors eventually acquire resistance to EGFR-TKI therapy. The most common mechanism of acquired resistance is a secondary mutation at exon 20, T790M gatekeeper mutation, occurring in approximately 50% of the NSCLC patients harboring EGFR mutations with acquired resistance to the TKIs (Gazdar, 2009). 

The EGFR mutations in NSCLC are primarily found in patients with adenocarcinoma, non-smokers, and females. Ethnic and demographic differences in the prevalence of these driver mutations have been previously reported, with low rates in white patients (~10–20%) and high rates in those of Asian descent (~40-50%) (Midha et al., 2015; Zhang et al., 2016). The prevalence of these mutations and the characteristics of NSCLC patients in the North African region have not been determined. Therefore, our objective in this study was to examine the EGFR mutation status in a sample group of Algerian NSCLC patients and its correlation with clinicopathological features.

## Materials and Methods

*Patients and samples*

This study was conducted at Ben-Badis University Hospital of Constantine between October 2015 and November 2018. It was carried out on 58 samples from unselected patients with histologically confirmed NSCLC who were mainly from the eastern Algerian regions. The formalin-fixed paraffin-embedded (FFPE) tissue samples prepared using biopsy or surgical specimens were obtained from Department of Anatomic Pathology. The available clinicopathological information, including patient age, gender, smoking status, and cancer staging, were gained from Department of Pneumology and Thoracic Surgery. 

*DNA extraction*

Genomic DNA was extracted from FFPE samples, 10-μm-thick tissue sections, using the AllPrep DNA/RNA FFPE Kit (Qiagen, Hidden, Germany) according to the manufacturer instructions. Then, it was quantified by NanoDrop 8000 Spectrophotometer (ThermoFisher, Waltham, USA).

*EGFR mutation analysis*

One hundred nanograms of DNA were amplified in a 25 μL reaction solution containing 12,5 μL of 2x AmpliTaq Gold 360 Master Mix with 0,5 μL of GC enhancer (Applied Biosystems, ThermoFisher, Waltham, USA), and 0.3μM of each primer pair for EGFR exons 19, 20 and 21 (Exon 19F: 5’-ATGTGGCACCATCTCACAATTGCC, 19R: 5’-CCACACAGCAAAGCAGA AACTCAC, Exon 20F: 5’-CATTCA TGCGTCTTCACCTG, 20R: 5’-CATATCCCCATGGCA AACTC, Exon 21F: 5’-GCTC AGAGCCTGGCATGAA, 21R: 5’-CATCCTCCCCTGCATGTGT).

The PCR conditions were as follows: initial denaturation at 95°C for 15 minutes, followed by 35 cycles of denaturation at 95°C for 30 seconds, annealing at 57°C for 30 seconds and extension at 72°C for 45 seconds, followed by a final extension step of 7 minutes at 72 °C. The PCR products were separated in a 2% agarose gel electrophoresis and further purified with a Purelink Quick Gel Extraction and PCR Purification Combo Kit (Invitrogen, ThermoFisher, Waltham, USA) according to the manufacturer’s instructions. The sequencing reaction was performed using the Big Dye Terminator V3.1 cycle sequencing kit (Applied Biosystems) with sequence-specific primers in both directions. Sequencing data were generated with the ABI-Prism 3500xl Genetic Analyzer and captured by the SeqScape software v3.0 (Applied Biosystems). Sequences were aligned to the EGFR GenBank reference sequence (NG_007726.3), and data were analyzed using Unipro UGENE software (v.34.0).

*Statistical analysis*

Data were analyzed using the GraphPad Prism software (version 7.0). Chi-square test was done to compare two groups. p <0.05 was considered as statistically significant. 

## Results

*Patients’ characteristics*

This study investigated 58 samples from patients with NSCLC, including 53 (91.4%) men and 5 (8.6%) women. The patients’ median age at diagnosis was 59 (ranging from 44 to 94 years old). They were histologically classified as 27 (46.5%) adenocarcinomas (ADC) and 14 (24%) non-adenomatous NCSCLs (non-ADC), which included 8 squamous cell carcinoma (SqCC) and 6 other subtypes. In addition, 9 (15.5%) cases were NCSCL favor ADC and 8 (14%) favor SqCC. The smoking history and clinical disease staging data were available for 40 (69%) and 48 (82.7%) patients, respectively. Twenty three of the patients (57.5%) were smokers and 17 (42.5%) nonsmokers. Regarding clinical stages of the disease, 27 (56%) cases were at the early-stage of disease (I-III) and 21 (44%) were at the advanced stage or metastasic (IV). 

*EGFR mutation status and its association with clinicopathological characteristics*

**Figure 1:**
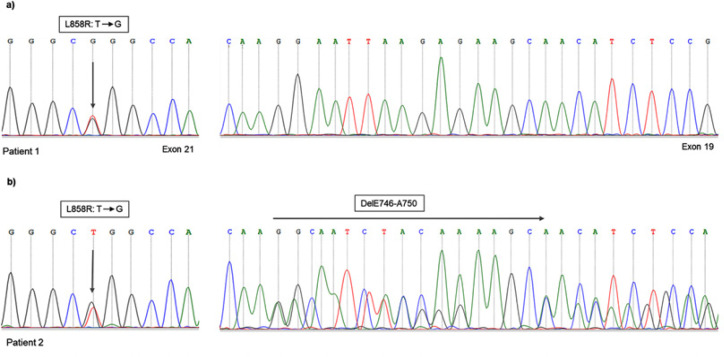
Examples of EGFR Mutation-Positive NSCLC Cases. (a) Patient 1, harboring L858R (Leu858Arg) missense mutation (T to G) in exon 21 with wild-type exon 19. (b) Patient 2, with dual mutations of L858R and in-frame deletion in exon 19 (DelE746-A750) mutations

Table 1 shows the clinicopathological characteristics of patients according to EGFR mutation status. We detected EGFR mutations in 23 out of 58 samples with an overall mutation rate of 39.6%. The median age of patients with EGFR-mutated was 58 (ranging from 47 to 73 years old). According to histological subtypes, a significantly higher incidence of EGFR mutation was observed in patients with confirmed ADC (14/27, 51.8%) compared to those with non-ADC (3/14, 21.4%; p=0.03). Moreover, a high mutation frequency was noted in patients with NSCLC favor SqCC 50% (4/8). Exon 21 L858R single mutation was detected in most patients harboring EGFR mutations, which represent 91.3% (21/23). The co-occurring of L858R and Ex19del (dual mutations) were identified in 2 (8.7%) cases. Both patients harboring dual mutations were diagnosed with ADC, one woman had L858R and DelL747_P753insS, and one man showed L858R and DelE746_A750 mutations (Figure 1).

Considering the clinical staging of cancer, early stages were significantly associated with a high frequency of EGFR mutations (14/27, 51.8%) compared to advanced stage (5/21, 23.8%; p=0.02). On the other hand, no significant relationship between EGFR mutation rate and age, gender, and smoking status was found. Furthermore, in subgroups of patients diagnosed with ADC, one (33.4%) of three women was harboring mutation and was nonsmoker, while among the evaluated male patients, EGFR mutations were detected in 13 (54%) of 24 of men, including 4/6 (66.6%) nonsmokers versus 6/14 (42.8%) smokers.

**Table 1:** EGFR Mutation Status and Clinicopathological Characteristics of Patients

	Overall N = 58 (100%)	Mutant N = 23 (39.6)	Wildtype N = 35 (60.4)	p-value
Age, year				
Median (range)	59 (44-94)	58 (47-73)	60 (44-94)	
< 60	28 (48.3)	12 (42.86)	16 (57.14)	0.29
≥ 60	28 (46.5)	10 (35.71)	18 (66.7)	
Gender				
Male	53 (91.4)	22 (41.5)	31 (58.5)	0.17
Female	5 (8.6)	1 (20)	4 (80)	
Smoking status				
Smoker	23 (39.6)	9 (39.1)	14 (60.9)	0.4
Non-smoker	17 (29.3)	6 (35.3)	11 (64.7)	
Unknown	18 (31.1)	8 (44.4)	10 (55.6)	
Histology				
ADC	27 (46.5)	14 (51.8)	13 (48.2)	0.03*
SqCC	8 (14)	2 (25)	6 (75)	
Favor ADC	9 (15.5)	2 (22.2)	7 (77.8)	
Favor SqCC	8 (14)	4 (50)	4 (50)	
Other types	6 (10)	1 (16.6)	5 (83.4)	
Disease Stage				
I - III	27 (46.5)	14 (51.85)	13 (48.15)	0.02*
IV	21 (36.2)	5 (23.81)	16 (76.19)	
Unspecified	10 (17.2)	4 (40)	6 (60)	

*, Statistically significant.

**Table 2:** The Frequency of EGFR Mutations Reported from the Regions of North-Africa and the Middle East

	Frequency (%)	N	Histology	Method	Study
North Africa					
Algeria	39.6	58	NSCLC	Sanger	This study
Morocco	21	120	ADC	TaqMan & Sanger	Errihani et al.
Tunisia	5.5	73	ADC	Sanger	Dhieb et al.
	44	50	ADC	IHC	Mraihi et al.
	11.5	26	ADC	ARMS/Scorpion	Arfaoui et al.
Middle East					
Gulf region	28.7	230	NSCLC	qPCR	Jazieh et al.
Levant region	15.6	205	ADC	ARMS/Scorpion	Tfayli et al.
Lebanon	11.9	201	Non-SqCC	ARMS/Scorpion	Naderi et al.
	8.5	106	ADC	ARMS/Scorpion	Fakhruddin et al.
Turkey	16.7	959	NSCLC	qPCR	Tezel et al.
	32.3	300	ADC	Pyrosequencing	Demiray et al.
	42.6	48	NSCLC	Sanger	Unal et al.

ARMS, Amplification-refractory mutation system; IHC, Immunohistochemistry; qPCR, Quantitative PCR.

## Discussion

The presence of EGFR mutations was shown to be associated with increased sensitivity to TKIs therapy in NSCLC (Lynch et al., 2004; Pao et al., 2004). Currently, EGFR-TKIs geﬁtinib and erlotinib are considered to be effective as first-line treatments for advanced NSCLC patients with EGFR-mutant (Inoue et al., 2013; Zhou et al., 2015). Furthermore, the incidence of EGFR mutations appears to be dependent on tumor histological type, gender, smoking history, and ethnic background (Midha et al., 2015; Zhang et al., 2016). In the present study, we investigated the EGFR mutation status in NSCLC patients from eastern Algeria. 

We found that the EGFR mutation rate was 39.6%, a frequency higher than that reported in Caucasian and quite similar to that in Asian patients. Varying results have been reported in ethnic groups closed to our population, such as North Africa and Middle East regions (Table 2). In contrast with our findings, Errihani et al., (2013) found a frequency of 21% regarding EGFR mutations among Moroccan patients with lung ADC. Besides, Dhieb et al., (2019) in Tunisia reported 5,5% EGFR mutations in patients with ADC. Similarly, Arfaoui et al., (2018) reported 11,5% EGFR mutations. Nevertheless, Mraihi et al., (2018) found a frequency of 44% in a cohort of Tunisian patients using mutation-specific immunohistochemistry, which was in line with our results. Studies conducted in Arab countries reported frequencies ranging from 8.5-12% for Lebanon (Fakhruddin et al., 2014; Naderi et al., 2015), 15,6% for the Levant region (Tfayli et al., 2017), and up to 28.7 for the Gulf region (Jazieh et al., 2015). In the Turkish population, a previous study by Unal et al., (2013) revealed EGFR mutation frequency of 42.6% in NSCLC patients from western Turkey, but in recent large-scale studies, Tezel et al., (2017) showed that the mutation rate in Turkish NSCLC patients was 16,7%. However, it was 32.3% in patients with ADC in a study by Demiray et al., (2018). These findings could support the heterogeneity in the prevalence of EGFR mutations. For example, reports from extensively evaluated populations also showed large variability with EGFR mutation frequencies in patients with lung ADC ranging between 6-41% in Europe, 3-42% in North America, and 20-76% in Asia-Pacific (Midha et al., 2015). Moreover, according to a meta-analysis of 456 studies conducted by Zhang et al., (2016), there was a significant heterogeneity in all analyzed variables related to the prevalence of EGFR mutations in NSCLC patients. Besides ethnic backgrounds, patient characteristics, clinical setting, and methodology may contribute to these differences. Thus, the data currently available do not enable us to arrive at a precise conclusion about this issue. 

Lung ADC histology represents one of the most important predictors for the testing and treatment of EGFR-mutant patients. EGFR mutations are primarily found in patients with ADC (~38%), and less frequent (~12%) in non-adenomatous NSCLCs (Zhang et al., 2016). In the same line, in our study, we found that EGFR mutations were more frequent in patients with ADC compared to those with confirmed non-ADC subtypes. Nonetheless, there was a high mutation frequency in NSCLC favor SqCC subtype. This finding may be explained by the fact that most positive cases tumor specimen originated from small biopsies. These results were consistent with previous findings by Ho et al., (2019) showing a high rate of mutations in small biopsy-diagnosed SqCC or favor SqCC, which may be adenosquamous carcinoma. Therefore, the testing of EGFR mutations could be recommended for patients with squamous NSCLC diagnosed from small biopsies.

Ex19del and L858R mutations represent approximately 47% and 41% of total EGFR mutations, respectively (Harrison et al., 2020). These two mutations have been reported to be associated with distinct prognostic implications in NSCLC and may require individualized treatment strategies (Liu et al., 2016; Kobayashi and Mitsudomi, 2016). Interestingly, we found frequencies pattern different from those reported in other studies, with the predominance of L858R single mutation and the presence of dual mutations of L858R/Ex19del. Although these two mutations lead to high sensitivity to EGFR-TKIs (Lynch et al., 2004; Pao et al., 2004; Riely et al., 2006), some studies demonstrated that patients with Ex19del mutation had a higher response and survival rates than those with L858R (Jackman et al., 2006; Wang et al., 2015; Jiang et al., 2019). However, in a meta-analysis done by Lee et al., (2015), there was a significantly longer progression-free survival (PFS) in patients harboring L858R than those with Ex19del mutation randomly assigned to chemotherapy. Furthermore, similar PFS benefit was observed in both mutations in NSCLC patients treated with TKI erlotinib in combination with VEGFR2 antagonist Ramucirumab (Nakagawa et al., 2019). On the other hand, dual mutations of L858R/Ex19del seem to be uncommon (Harrison et al., 2020), and the response of this subset of patients to EGFR-TKIs compared with those with a single mutation remains controversial (Zhang et al., 2007; Wei et al., 2014; Peng et al., 2018). 

Regarding the relationship between EGFR mutations and patients’ clinical characteristics, data from the literature revealed that EGFR mutations were more frequent in never-smokers and female patients (Ren et al., 2012; Zhang et al., 2016; Tseng et al., 2017). In contrast to several reports, the evaluation of available clinical data in our patients showed no correlation between EGFR mutations and women and non-smoking status. Though, we found that patients diagnosed at early stage of the disease exhibited higher EGFR mutation frequency compared with those with metastatic or at advanced stage. This may due to the quality of the specimen, as most of our patients at advanced stage were diagnosed with small biopsies. 

Sanger sequencing remains the gold standard method to detect EGFR mutations, preferably rare and unknown mutations, but it has a relatively low sensitivity and more suitable for detecting mutations in surgical specimens (Liang et al., 2018). Moreover, considering the high recurrence rate in NSCLC patients at stages II and III (Sasaki et al., 2014), testing EGFR mutations in patients at early stage would be useful to pave the way toward more effective therapeutic strategies. Although, some subgroups of NSCLC patients are more likely harboring mutations such as Asian females, never-smokers, with ADC, and Caucasian non-smokers (Zhang et al., 2016), the EGFR mutation testing is recommended by different organizations’ guidelines (CAP, AMP, IASLC, and ESMO) for all patients with advanced nonsquamous NSCLC regardless of clinical characteristics, including some patients with SqCC such as non-smokers or youngers (Pennell et al., 2019).

This study included some limitations. First, the sample size was relatively small, especially the scarce number of female patients. Second, some patients did not have clinical data (i.e. smoking and stage of the disease) . Finally, the patients involved in our study were mostly from the Eastern region of Algeria. Despite the limitations, our results may predict a high benefit for Algerian NSCLC patients from EGFR-TKI therapy and help to select patients eligible for future research, particularly prospective investigations on the response of patients with EGFR mutation to TKI-based treatments.

In conclusion, our study suggested that approximately 40% of Algerian NSCLC patients would harbor EGFR mutations and therefore would be eligible for TKI-based therapy. The observed EGFR mutation frequency was quite similar to that found in Asian population rather than Caucasian population. These findings demonstrated the need to implement reflex EGFR testing in daily clinical practice. . We suggest patients with NSCLC to be tested for EGFR mutations, especially those with adenocarcinoma regardless of clinical characteristics. Nonetheless, these preliminary data require further investigations on a large number of patients. 

## Ethics approval

 This study was conducted according to the international guideline of the Helsinki Declaration (1964) and our national legislation for medical research ethics.

## Conflicts of interest/Competing interests

The authors declare that there are no conflicts of interest. 
